# Stability of filament-wound hyperbolic flexible pipes under internal pressure based on non-geodesic winding

**DOI:** 10.1038/s41598-021-85326-y

**Published:** 2021-03-15

**Authors:** Guo-min Xu, Chang-geng Shuai

**Affiliations:** 1 Institute of Noise and Vibration, Naval University of Engineering, Wuhan, 430033 China; 2 National Key Laboratory on Ship Vibration and Noise, Wuhan, 430033 China

**Keywords:** Civil engineering, Mechanical engineering, Composites

## Abstract

Filament-wound flexible pipes are widely used to transport fluid in pipeline systems, proved extremely useful in marine engineering. The hyperbolic flexible pipes have good vibration suppression performance, but they are easily deformed under internal pressure. This paper focuses on the stability of hyperbolic flexible pipes based on the composite Reissner shell theory and the transfer-matrix method. The nonlinear stretch of the reinforced filament and the fiber bridge effect are considered in the model. The calculation results show that a large winding angle reduces the deformation and the meridional stress. The available initial winding angle is limited by the geometry and the slippage coefficient of flexible pipe. The reinforced filament of high tensile modulus will reduce the deformation of the pipe. Compared with the geodesic winding trajectory, non-geodesic winding trajectories improves the stability of the pipe. The theoretical result is verified by the finite element analysis. The investigation method and results present in this paper will guide the design and optimization of more novel flexible pipes in the future.

## Introduction

Filament-wound flexible pipes are widely used to transport the fluid at locations requiring flexible connection in pipeline systems. They protect the pipeline from damage caused by mechanical vibration and shock. Compared with metal flexible pipes, the fiber-reinforced flexible pipes have many advantages, such as light weight, large displacement compensation, and high vibration suppression, which proved extremely useful in marine engineering. Flexible pipes are filament/rubber composites compounded by the inner rubber layer, reinforced layer, and outer rubber layer. The reinforced layer is fabricated with the filament winding technique. Hyperbolic flexible pipe is regarded as a novel category of flexible pipes, owning lower natural frequency compared with common cylindrical and spherical pipes^1^. So hyperbolic flexible pipes will have better vibration suppression performance and broader applications in the future. However, hyperbolic flexible pipes are easily deformed under internal pressure, since they have negative Gaussian curvature. Large deformation will decrease the reliability of the pipe significantly. Therefore, the stability of hyperbolic flexible pipes subject to internal pressure needs to be investigated carefully.

Recent research has shown that the stability of flexible pipes could be achieved by optimal design of filament-winding trajectory. The deformation of cylindrical flexible pipes under internal pressure has been studied in depth based on membrane theory^2^, classical laminated shell theory^3–5^, and three-dimensional elastic theory^6,7^. They have reached a consistent conclusion that the optimal winding angle for stable cylindrical flexible pipes under internal pressure is near 55 degrees. The optimal winding angle will drift between 52 and 55 degrees considering the non-negligible elongation of reinforced fibers. The optimal winding angle was verified by finite element analysis and experiments^8–10^. Some investigations have focused on filament winding on non-cylindrical surfaces. Zhang et al.^11^ investigated the stability of filament-wound spherical flexible pipes under internal pressure. Winding angle deviating from the optimal value will cause significant axial deformation for spherical flexible pipes. Zu et al.^12^ proposed a design method for small-angle winding of composite square pipes with the constraint of non-slippage. Hu et al.^13^ proposed a design method for toroidal pipes based on geodesic winding. The winding angle is decreasing with the winding angle moving from the outer to the inner periphery. It should be noted that geodesic winding is not very suitable for non-cylindrical flexible pipes. Although geodesic trajectory shows great winding stability on curved surfaces, the trajectory is simply determined with a given mandrel and initial winding angle, restricting the available distribution of winding angle. The winding trajectory, however, has to match the direction of the major principal stress to achieve the stability of the flexible pipes under internal pressure^14^. So it is more desirable to optimize winding trajectory based on non-geodesic winding. Filament winding on doubly-curved shells of revolution has gained increasing attention in recent years. Koussios et al.^15^ established first-order differential equations describing non-geodesic trajectories on an ellipsoid, cone, and sphere mathematically, but the external load was not considered in the model. Min et al.^16^ proposed a topological mapping algorithm for optimal winding trajectory on an elliptical shell. Zu et al.^17^investigated the distribution of winding angle on a conical shell, concluding that the winding angle distribution has an increase with the increase of the slippage coefficient or the initial winding angle. However, investigations on filament winding on hyperboloids are very limited. Wan et al.^18^ mathematically calculated the geodesic trajectory on a hyperbolic surface. The deformation of filament-wound hyperbolic pipe was not considered in the paper.

As already known from the research above mentioned, the stability of filament-wound flexible pipes has gained sufficient attention. Nevertheless, the stability of hyperbolic flexible pipes needs more investigation. Firstly, the research on non-geodesic winding on the hyperboloid is very limited. It is needed to propose a model of non-geodesic winding trajectory to study the deformation of the hyperbolic flexible pipe. Flexible pipes are fiber/rubber composites with much smaller elastic modulus compared with common resin composites. The mismatch between geodesic winding trajectory and stress distribution induced by internal pressure will cause severe deformation on the flexible section. Secondly, the non-geodesic trajectory has to avoid the fiber bridge effect on the section of negative Gaussian curvature, resulting in additional constraints on the design of the winding trajectory. Thirdly, the stretch of reinforced fiber needs careful consideration. The winding angle will change under different internal pressure because of the non-negligible stretch of reinforced fibers in rubber-based composites, while the winding angle remains unchanged in resin-based composites. The variation of winding angle will lead to the variation of the anisotropy of the reinforced layer.

In this paper, the stability of filament-wound hyperbolic flexible pipes is studied based on non-geodesic winding. The deformation of the flexible pipe under internal pressure is investigated based on the composite Reissner shell theory and transfer-matrix method. The transfer-matrix equations are solved by the precise integration method^19,20^. The nonlinear variance of the winding angle due to the stretch of reinforced fiber is considered in the theoretical model. A finite element model is established to verify the theoretical optimization results based on the software CADWIND and ABAQUS. The investigation method and results present in this paper will guide the design and optimization of hyperbolic flexible pipes in the future.

## Methods

### Non-geodesic winding trajectory on hyperbolic flexible pipes

**Figure 1:**
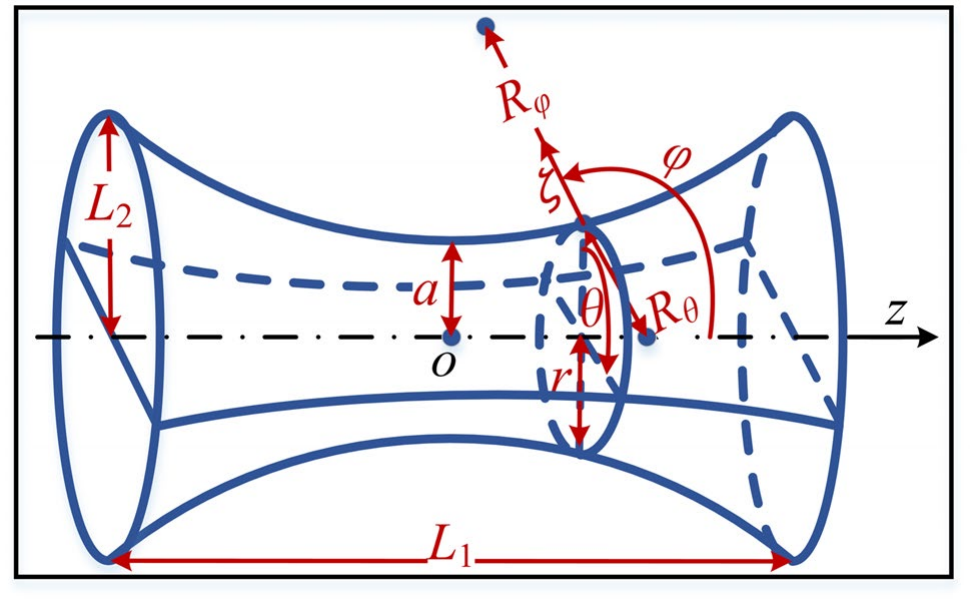
The geometry of hyperbolic flexible pipes.

The geometry of hyperbolic flexible pipes is shown in Fig. 1. Cylindrical coordinate system (*r, θ, z*) and curvilinear coordinate system (*φ, θ, ζ*) are established. Point *o* is the origin of the cylindrical coordinate system. Correspondingly, the deformation in the meridional (*φ*), circumferential (*θ*) and normal directions (*ζ*) are represented by symbols *u*, *v* and *w*. *R*_*φ*_ and *R*_*θ*_ are the principal radius of curvature of flexible pipes. *r* denotes the distance from the point on the meridian to the axis of rotation.

The equations of the hyperboloid of one sheet at the curvilinear coordinate system are written as^21^

1 $$\begin{array}{*{20}l} {R_{\varphi } = \frac{{ - a^{2} b^{2} }}{{\sqrt {(a^{2} \sin^{2} \varphi - b^{2} \cos^{2} \varphi )^{3} } }}} \hfill \\ {R_{\theta } = \frac{{a^{2} }}{{\sqrt {a^{2} \sin^{2} \varphi - b^{2} \cos^{2} \varphi } }}} \hfill \\ \end{array}$$

where a and b are the lengths of semi-transverse and semi-conjugate axes of the meridian. Specially,

2 $$b = \frac{{aL_{1} }}{{{2}\sqrt {L_{2}^{2} - a^{2} } }}$$

where *L*_*1*_ and *L*_*2*_ are annotated in Fig. 1. With Eq. (1), the parametric equation of the hyperboloid of one sheet at the curvilinear coordinate system is easily written as

3 $${\mathbf{S}}(\theta ,\varphi ) = \left( {\begin{array}{*{20}c} {r\cos \theta } \\ {r\sin \theta } \\ {\frac{{{\text{sign}} (\varphi - \frac{\pi }{2})b^{2} }}{{\sqrt {a^{2} \tan^{2} \varphi - b^{2} } }}} \\ \end{array} } \right)$$

where $$r={R}_{\theta }sin\varphi$$.

The parametric equation of the hyperboloid of one sheet at the cylindrical coordinate system is easily written as

4 $${\mathbf{S}}(\theta ,z) = \left( {\begin{array}{*{20}c} {\rho (z)\cos \theta } \\ {\rho (z)\sin \theta } \\ z \\ \end{array} } \right)$$

where

5 $$\rho (z) = a\sqrt {1 + \frac{{z^{2} }}{{b^{2} }}}$$

The differential equation of non-geodesic trajectories is written as^15^

6 $$\frac{d\sin \alpha }{{dz}} = - \frac{1}{2E}\frac{dE}{{dz}}\sin \alpha \pm \lambda \sqrt G [(k_{p} - k_{m} )\sin^{2} \alpha + k_{m} ]$$

where *α* is the winding angle and *λ* is the slippage coefficient. The coefficients E and G are related to the metrics on the surface

7 $$\begin{array}{*{20}l} {E = (\frac{{\partial {\mathbf{S}}}}{\partial \theta })^{2} = a^{2} (1 + \frac{{z^{2} }}{{b^{2} }})} \hfill \\ {G = (\frac{{\partial {\mathbf{S}}}}{\partial z})^{2} = 1 + \frac{{a^{2} z^{2} }}{{b^{4} + b^{2} z^{2} }}} \hfill \\ \end{array}$$

The two principal curvatures at the cylindrical coordinate system are written as

8 $$\begin{array}{*{20}l} {k_{m} = \frac{a}{{b^{2} (\frac{{(a^{2} + b^{2} )z^{4} + (2b^{4} + a^{2} b^{2} )z^{2} + b^{6} }}{{b^{4} z^{2} + b^{6} }})^{\frac{3}{2}} }}} \hfill \\ {k_{p} = - \frac{1}{{a\sqrt {1 + \frac{{z^{2} }}{{b^{2} }}} \sqrt {1 + \frac{{a^{2} z^{2} }}{{b^{4} + b^{2} z^{2} }}} }}} \hfill \\ \end{array}$$

It should be noted that the non-geodesic trajectories will transform to the geodesic trajectory when the slippage coefficient *λ* = 0. The geodesic trajectory on the hyperboloid of one sheet is written as

9 $$a\sqrt {z^{2} + b^{2} } \sin \alpha = {\text{const}}$$

Equation (9) shows that the geodesic trajectory is determined with given geometry of the flexible pipes and initial winding angle. The stability can hardly be achieved with geodesic winding since the winding angle is required to match the direction of the major principal stress.

In order to avoid the fiber bridge on the surface of negative Gaussian curvature, the following constraint of winding angle has to be satisfied^22^:

10 $$\tan^{2} \alpha + \frac{{R_{\theta } }}{{R_{\varphi } }} \ge 0$$

### Deformation of hyperbolic flexible pipes under internal pressure

The mechanical model of the flexible pipes is established based on the composite Reissner shell theory. The kinematic equations and equilibrium equations are simplified because of the axial symmetry of the internal pressure. The kinematic equations are simplified as

11 $$\begin{array}{*{20}l} {\varepsilon_{\varphi } = \frac{1}{{R_{\varphi } }}(\frac{du}{{d\varphi }} + w)} \hfill \\ {\varepsilon_{\theta } = \frac{1}{r}(u\cos \varphi + w\sin \varphi )} \hfill \\ {\psi_{\varphi } = \frac{1}{{R_{\varphi } }}(u - \frac{dw}{{d\varphi }})} \hfill \\ {\chi_{\varphi } = \frac{1}{{R_{\varphi } }}\frac{{d\psi_{\varphi } }}{d\varphi }} \hfill \\ {\chi_{\theta } = \frac{{\psi_{\varphi } \cos \varphi }}{r}} \hfill \\ \end{array}$$

where ${\varepsilon }_{\varphi }, {\varepsilon }_{\theta }$ are the normal strains and ${\chi }_{\varphi }, {\chi }_{\theta }$ are the curvature changes. The symbol ${\psi }_{\varphi }$ represents the angle between the surface normal before and after the surface deformation.

The equilibrium equations are simplified as

12 $$\begin{array}{*{20}l} {\frac{{dN_{\varphi } }}{d\varphi } + \frac{{R_{\varphi } \cos \varphi }}{r}(N_{\varphi } - N_{\theta } ) + Q_{\varphi } = 0} \hfill \\ {\frac{{dQ_{\varphi } }}{{R_{\varphi } d\varphi }} + \frac{\cos \varphi }{r}Q_{\varphi } - \frac{{N_{\varphi } }}{{R_{\varphi } }} - \frac{{N_{\theta } \sin \varphi }}{r} = - q_{\zeta } } \hfill \\ {\frac{{dM_{\varphi } }}{d\varphi } + \frac{{R_{\varphi } \cos \varphi }}{r}(M_{\varphi } - M_{\theta } ) - R_{\varphi } Q_{\varphi } = 0} \hfill \\ \end{array}$$

where ${N}_{\varphi }, {N}_{\theta }$ are the normal force resultants and ${M}_{\varphi }, {M}_{\theta }$ are the bending moment resultants. The symbol ${Q}_{\varphi }$ represents the transverse shear force resultants.

The reinforced layers of flexible pipes are fabricated with synthetic fibers such as aramid or nylon. The elastic modulus and strength of the synthetic fibers are much greater than rubber materials. Therefore, only the reinforced layer is considered in the mechanical analysis, while the influence of the inner and outer rubber layers is neglected. The single-layer lamina in the reinforced layer is fabricated with two layers of cross-wound filament. The axisymmetric stress–strain relations of the *k*th lamina in the reinforced layer are written as

13 $$\left[ {\begin{array}{*{20}c} {\sigma_{\varphi } } \\ {\sigma_{\theta } } \\ {0} \\ \end{array} } \right]_{k} = \left[ {\begin{array}{*{20}c} {\overline{Q}_{11}^{k} } & {\overline{Q}_{12}^{k} } & 0 \\ {\overline{Q}_{12}^{k} } & {\overline{Q}_{22}^{k} } & 0 \\ 0 & 0 & {0} \\ \end{array} } \right]\left[ {\begin{array}{*{20}c} {\varepsilon_{\varphi } } \\ {\varepsilon_{\theta } } \\ {0} \\ \end{array} } \right]_{k}$$

where ${\bar{Q}}_{ij}^{k}$ represent the off-axis stiffness coefficients of the *k*th lamina, which are written as^23^

14 $${\overline{\mathbf{Q}}}_{k} = \frac{1}{2}[{\mathbf{H}}_{k} (\alpha ){\mathbf{Q}}_{k} {\mathbf{H}}_{k}^{{\text{T}}} (\alpha ) + {\mathbf{H}}_{{\text{k}}} ( - \alpha ){\mathbf{Q}}_{k} {\mathbf{H}}_{k}^{{\text{T}}} ( - \alpha )]$$

where ${Q}_{ij}^{k}$ represent the material constants of the *k*th orthotropic lamina. The fiber coordinates of the lamina are written as 1 and 2, where direction 1 is parallel to the fibers and 2 is perpendicular to them. The ${Q}_{ij}^{k}$ are defined as

15 $$\begin{array}{*{20}c} {Q_{11}^{k} = \frac{{E_{1}^{k} }}{{1 - \mu_{12}^{k} \mu_{21}^{k} }},} & {Q_{12}^{k} = \frac{{\mu_{12}^{k} E_{1}^{k} }}{{1 - \mu_{12}^{k} \mu_{21}^{k} }},} & {Q_{22}^{k} = \frac{{E_{2}^{k} }}{{1 - \mu_{12}^{k} \mu_{21}^{k} }}} \\ \end{array}$$

where ${E}_{1}^{k}$ and ${E}_{2}^{k}$ are elasticity modulus of the *k*th lamina in the 1 and 2 directions, respectively; ${\mu }_{12}^{k}$ is the major Poisson’s ratio. ${\mu }_{21}^{k}$ is determined by the equation ${\mu }_{21}^{k}{E}_{2}^{k}={\mu }_{12}^{k}{E}_{1}^{k}$. The transformation matrix ***H***_*k*_ is written as

16 $${\mathbf{H}}_{k} = \left[ {\begin{array}{*{20}c} {\cos^{2} \alpha_{k} } & {\sin^{2} \alpha_{k} } & { - 2\sin \alpha_{k} \cos \alpha_{k} } \\ {\sin^{2} \alpha_{k} } & {\cos^{2} \alpha_{k} } & {2\sin \alpha_{k} \cos \alpha_{k} } \\ {\sin \alpha_{k} \cos \alpha_{k} } & { - \sin \alpha_{k} \cos \alpha_{k} } & {\cos^{2} \alpha_{k} - \sin^{2} \alpha_{k} } \\ \end{array} } \right]$$

where winding angle *α*_*k*_ is the angle between direction 1 of the fiber and the direction $\varphi$ of the curvilinear coordinate system.

The stresses over the shell thickness are integrated to obtain the force and moment resultants, which are

17 $$\left[ {\begin{array}{*{20}c} {N_{\varphi } } \\ {N_{\theta } } \\ \end{array} } \right]\begin{array}{*{20}l} {\begin{array}{*{20}c} { = \int_{{ - {h \mathord{\left/ {\vphantom {h 2}} \right. \kern-\nulldelimiterspace} 2}}}^{{{h \mathord{\left/ {\vphantom {h 2}} \right. \kern-\nulldelimiterspace} 2}}} {\left[ {\begin{array}{*{20}c} {\sigma_{\varphi } } \\ {\sigma_{\theta } } \\ \end{array} } \right]d\zeta } } & {\left[ {\begin{array}{*{20}c} {M_{\varphi } } \\ {M_{\theta } } \\ \end{array} } \right] = \int_{{ - {h \mathord{\left/ {\vphantom {h 2}} \right. \kern-\nulldelimiterspace} 2}}}^{{{h \mathord{\left/ {\vphantom {h 2}} \right. \kern-\nulldelimiterspace} 2}}} {\left[ {\begin{array}{*{20}c} {\sigma_{\varphi } } \\ {\sigma_{\theta } } \\ \end{array} } \right]\zeta d\zeta } } \\ \end{array} } \hfill \\ \end{array}$$

where *N*_*φ*_, *N*_*θ*_ are the normal force resultants; *M*_*φ*_*, M*_*θ*_ are the bending moment resultants. Substituting Eqs. (13) into Eqs. (17) and performing the integration over the thickness, yields

18 $$\left[ {\begin{array}{*{20}c} {N_{\varphi } } \\ {N_{\theta } } \\ {M_{\varphi } } \\ {M_{\theta } } \\ \end{array} } \right] = \left[ {\begin{array}{*{20}c} {A_{11} } & {A_{12} } & {0} & {0} \\ {A_{12} } & {A_{22} } & {0} & {0} \\ {0} & {0} & {D_{11} } & {D_{12} } \\ {0} & {0} & {D_{12} } & {D_{22} } \\ \end{array} } \right]\left[ {\begin{array}{*{20}c} {\varepsilon_{\varphi } } \\ {\varepsilon_{\theta } } \\ {\chi_{\varphi } } \\ {\chi_{\theta } } \\ \end{array} } \right]$$

where the stiffness coefficients *A*_*ij*_*, D*_*ij*_ are given as

19 $$\left( {A_{ij} ,D_{ij} } \right) = \sum\limits_{k = 1}^{N} {\int_{{\zeta_{k - 1} }}^{{\zeta_{k} }} {\left( {1,\zeta^{2} } \right)\overline{Q}_{ij}^{k} d\zeta } }$$

Introduce the state vector

20 $$\left\{ {\mathbf{Z}} \right\} = \left\{ {\begin{array}{*{20}l} u \hfill & w \hfill & {\psi_{\varphi } } \hfill & {N_{\varphi } } \hfill & {Q_{\varphi } } \hfill & {M_{\varphi } } \hfill \\ \end{array} } \right\}^{{\text{T}}}$$

In order to use transfer-matrix method, Eqs. (11), (12) and (18) can be written as

21 $$\frac{d}{ds}\left\{ {\mathbf{Z}} \right\} = {\mathbf{A}}\left\{ {\mathbf{Z}} \right\} - \left\{ {\mathbf{q}} \right\}$$

where $$\left\{{\varvec{q}}\right\}={\left\{\begin{array}{ccc}\begin{array}{cc}0& 0\end{array}& \begin{array}{cc}0& 0\end{array}& \begin{array}{cc}p& 0\end{array}\end{array}\right\}}^{T}$$ and $ds={R}_{\varphi }d\varphi$. Rewrite Eq. (21) as

22 $$\frac{d}{ds}\left\{ {{\tilde{\mathbf{Z}}}} \right\} = {\mathbf{B}}\left\{ {{\tilde{\mathbf{Z}}}} \right\}$$

where extended state vector $$\stackrel{\sim }{{\varvec{Z}}}={\left[\begin{array}{cc}{\varvec{Z}}& 1\end{array}\right]}^{T}$$. The matrix ***B*** is written as

23 $${\mathbf{B}} = \left[ {\begin{array}{*{20}l} {\frac{{S_{33} \cos \varphi }}{r}} \hfill & {(\frac{{S_{33} \sin \varphi }}{r} - \frac{1}{{R_{\varphi } }})} \hfill & {\frac{{S_{34} \cos \varphi }}{r}} \hfill & {S_{31} } \hfill & 0 \hfill & {S_{32} } \hfill & 0 \hfill \\ {\frac{1}{{R_{\varphi } }}} \hfill & 0 \hfill & { - 1} \hfill & 0 \hfill & 0 \hfill & 0 \hfill & 0 \hfill \\ {\frac{{S_{43} \cos \varphi }}{r}} \hfill & {\frac{{S_{43} \sin \varphi }}{r}} \hfill & {\frac{{S_{44} \cos \varphi }}{r}} \hfill & {S_{41} } \hfill & 0 \hfill & {S_{42} } \hfill & 0 \hfill \\ {\frac{{S_{13} \cos^{2} \varphi }}{{r^{2} }}} \hfill & {\frac{{S_{13} \cos \varphi \sin \varphi }}{{r^{2} }}} \hfill & {\frac{{S_{14} \cos^{2} \varphi }}{{r^{2} }}} \hfill & {\frac{{(S_{11} - 1)\cos \varphi }}{r}} \hfill & { - \frac{1}{{R_{\varphi } }}} \hfill & {\frac{{S_{12} \cos \varphi }}{r}} \hfill & 0 \hfill \\ {\frac{{S_{13} \cos \varphi \sin \varphi }}{{r^{2} }}} \hfill & {\frac{{S_{13} \sin^{2} \varphi }}{{r^{2} }}} \hfill & {\frac{{S_{14} \cos \varphi \sin \varphi }}{{r^{2} }}} \hfill & {(\frac{1}{{R_{\varphi } }} + \frac{{S_{11} \sin \varphi }}{r})} \hfill & { - \frac{\cos \varphi }{r}} \hfill & {\frac{{S_{12} \sin \varphi }}{r}} \hfill & { - q_{\zeta } } \hfill \\ {\frac{{S_{23} \cos^{2} \varphi }}{{r^{2} }}} \hfill & {\frac{{S_{23} \cos \varphi \sin \varphi }}{{r^{2} }}} \hfill & {\frac{{S_{24} \cos^{2} \varphi }}{{r^{2} }}} \hfill & {\frac{{S_{21} \cos \varphi }}{r}} \hfill & 1 \hfill & {\frac{{(S_{22} - 1)\cos \varphi }}{r}} \hfill & 0 \hfill \\ 0 \hfill & 0 \hfill & 0 \hfill & 0 \hfill & 0 \hfill & 0 \hfill & 0 \hfill \\ \end{array} } \right]$$

where

24 $$\begin{array}{*{20}l} {\begin{array}{*{20}c} {S_{11} = \frac{{B_{11} B_{12} - A_{11} D_{12} }}{{B_{11}^{2} - A_{11} D_{11} }},} & {S_{12} = \frac{{A_{12} B_{11} - A_{11} B_{12} }}{{B_{11}^{2} - A_{11} D_{11} }},} \\ \end{array} } \hfill \\ {S_{13} = \frac{{D_{11} A_{12}^{2} - 2A_{12} B_{11} B_{12} + A_{22} B_{11}^{2} + A_{11} B_{12}^{2} - A_{11} A_{22} D_{11} }}{{B_{11}^{2} - A_{11} D_{11} }},} \hfill \\ {S_{14} = - \frac{{B_{11} B_{12}^{2} - B_{11}^{2} B_{22} - A_{11} B_{12} D_{12} + A_{12} B_{11} D_{12} - A_{12} B_{12} D_{11} + A_{11} B_{22} D_{11} }}{{B_{11}^{2} - A_{11} D_{11} }},} \hfill \\ {\begin{array}{*{20}c} {S_{21} = \frac{{B_{11} D_{12} - B_{12} D_{11} }}{{B_{11}^{2} - A_{11} D_{11} }},} & {S_{22} = \frac{{B_{11} B_{12} - A_{11} D_{12} }}{{B_{11}^{2} - A_{11} D_{11} }},} & {S_{23} = S_{14} ,} \\ \end{array} } \hfill \\ {S_{24} = \frac{{D_{22} B_{11}^{2} - 2B_{11} B_{12} D_{12} + D_{11} B_{12}^{2} + A_{11} D_{12}^{2} - A_{11} D_{11} D_{22} }}{{B_{11}^{2} - A_{11} D_{11} }},} \hfill \\ {\begin{array}{*{20}c} {S_{31} = - \frac{{D_{11} }}{{B_{11}^{2} - A_{11} D_{11} }},} & {S_{32} = \frac{{B_{11} }}{{B_{11}^{2} - A_{11} D_{11} }},} & {S_{42} = - \frac{{A_{11} }}{{B_{11}^{2} - A_{11} D_{11} }},} \\ \end{array} } \hfill \\ {\begin{array}{*{20}l} {S_{33} = - S_{11} ,} \hfill & {S_{34} = - S_{21} ,} \hfill & {S_{41} = S_{32} ,} \hfill & {S_{43} = - S_{12} ,} \hfill & {S_{44} = - S_{22} } \hfill \\ \end{array} } \hfill \\ \end{array}$$

The solution of Eq. (22) is

25 $$\left\{ {{\tilde{\mathbf{Z}}}} \right\}_{s2} = \exp ({\mathbf{B}}_{s1} \delta_{s} )\left\{ {{\tilde{\mathbf{Z}}}} \right\}_{{s{1}}} = {\mathbf{T}}_{s1} \left\{ {{\tilde{\mathbf{Z}}}} \right\}_{{s{1}}}$$

where ***T***_s1_ is the transfer matrix of extended state vector of two adjacent sections *s*_1_ and s_2_ along the meridional direction. The transfer matrix ***T***_s1_ is calculated by the precise integration method^19^. The stress and strain distribution of the reinforced layer under internal pressure will be obtained with Eq. (25) and boundary conditions.

**Figure 2:**
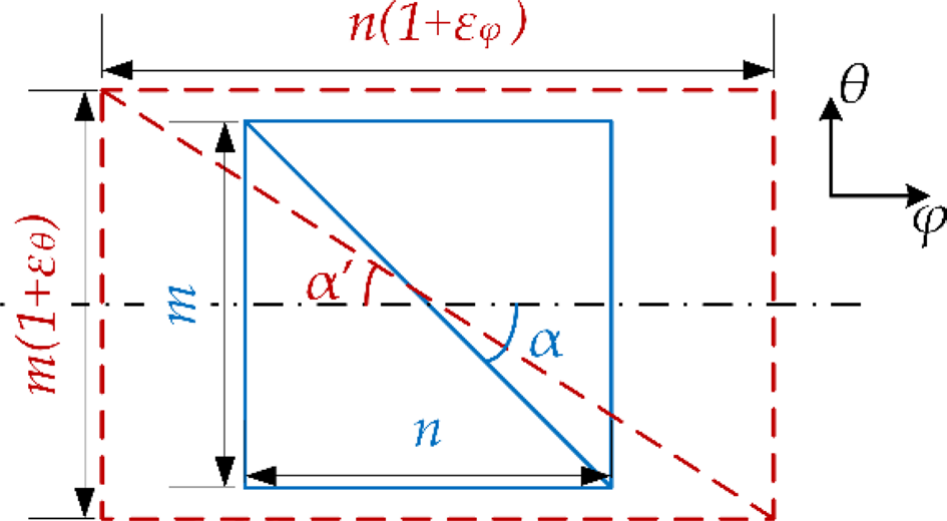
The slight change of winding angle under high internal pressure.

Because of the small elastic modulus of the rubber, the stretch of reinforced fiber will cause a slight change of the winding angle and the anisotropy of the reinforced layer under high internal pressure, as shown in Fig. 2. So the stretch of reinforced fiber is not negligible. Considering the strains of the reinforced layer, the updated winding angle and the transformation matrix of the stiffness matrix are written as

26 $$\begin{array}{*{20}c} {\tan \alpha^{\prime} = \frac{{1 + \varepsilon_{\theta } }}{{1 + \varepsilon_{\varphi } }}\tan \alpha ,} & {{\mathbf{H^{\prime}}}_{k} = \left[ {\begin{array}{*{20}c} {\cos^{2} \alpha^{\prime}_{k} } & {\sin^{2} \alpha^{\prime}_{k} } & { - 2\sin \alpha^{\prime}_{k} \cos \alpha^{\prime}_{k} } \\ {\sin^{2} \alpha^{\prime}_{k} } & {\cos^{2} \alpha^{\prime}_{k} } & {2\sin \alpha^{\prime}_{k} \cos \alpha^{\prime}_{k} } \\ {\sin \alpha^{\prime}_{k} \cos \alpha^{\prime}_{k} } & { - \sin \alpha^{\prime}_{k} \cos \alpha^{\prime}_{k} } & {\cos^{2} \alpha^{\prime}_{k} - \sin^{2} \alpha^{\prime}_{k} } \\ \end{array} } \right]} \\ \end{array}$$

The deformation of flexible pipes can be easily calculated with the following formula

27 $${\text{Deformation}} = \sqrt {u^{2} + w^{2} }$$

**Figure 3:**
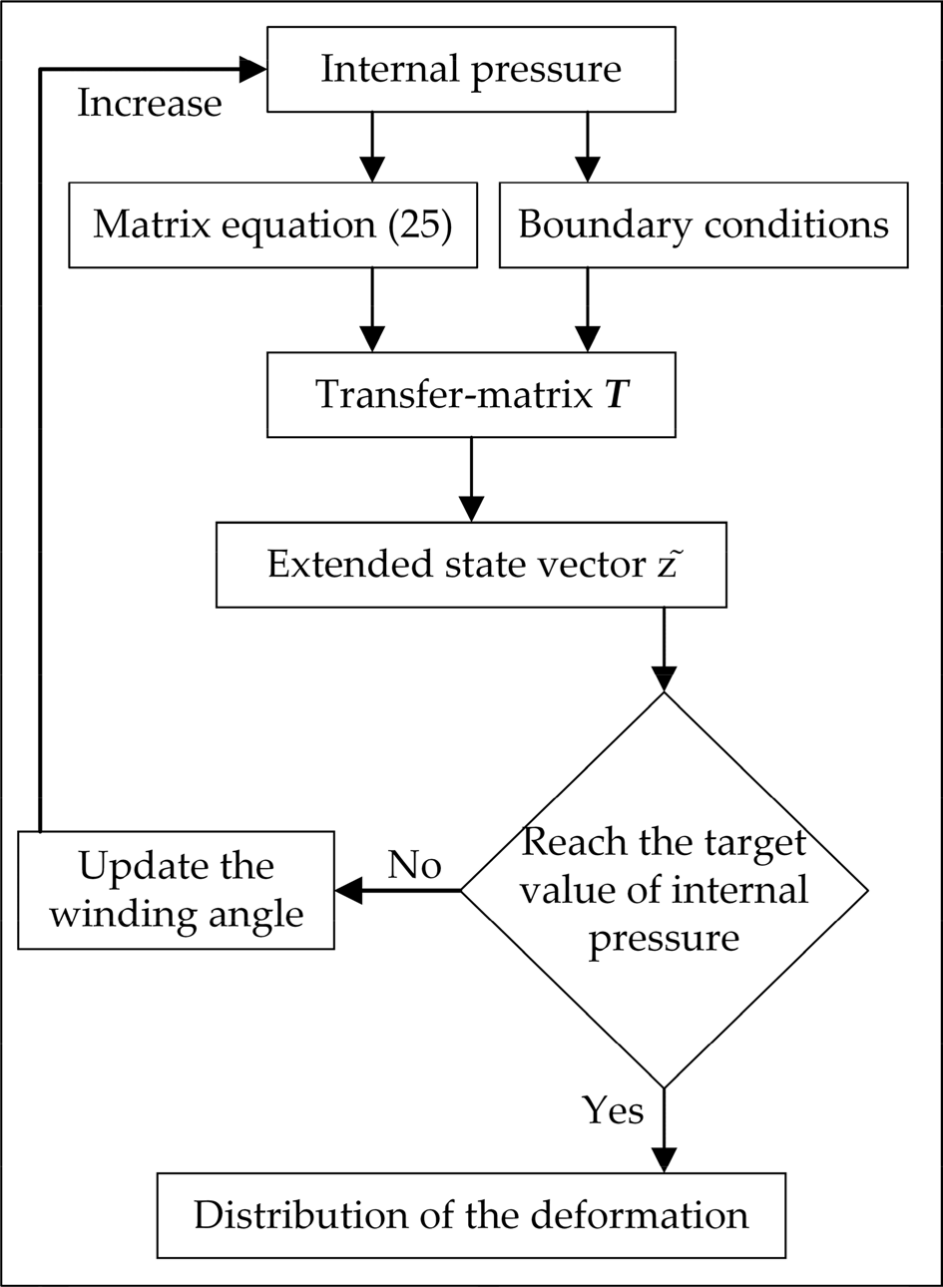
The approach of deformation calculation of fialment-wound hyperbolic flexible pipes.

The approach of deformation calculation is shown in Fig. 3. The increase of internal pressure is divided into many small steps. The winding angle and anisotropy of the reinforced layer will be updated in each step.

### The finite element model of filament-wound hyperbolic flexible pipes

**Figure 4:**
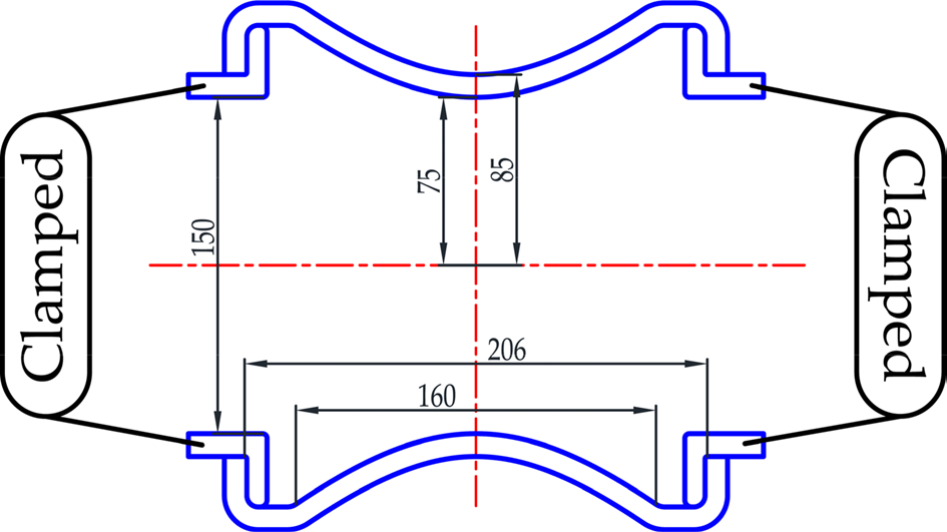
The geometry and boundary condition of calculated hyperbolic flexible pipe. The unit of length in the figure is millimeter.

The finite element model is established to simulate the deformation of hyperbolic flexible pipes and verify the results of theoretical calculation. The geometry of calculated DN150 hyperbolic flexible pipe is shown in Fig. 4. The flexible section is fabricated with neoprene and aramid filament whose elastic properties are listed in Table 1.

**Table 1:** Typical properties of materials.

Type of aramid filament	1100dtex
Elongation at Break (%)	3.2
Tensile modulus (GPa)	49.7
Tensile strength (MPa)	1591
Slippage coefficient	0.15
Elastic modulus of neoprene (MPa)	6
Thickness of the reinforced layer (mm)	1.2

The boundary conditions in both mathematical and FE models are clamped–clamped.

28 $$\begin{array}{*{20}c} {\left. u \right|_{{z = - \frac{{L_{1} }}{2}}} = 0} & {\left. u \right|_{{z = \frac{{L_{1} }}{2}}} = 0} \\ {\left. w \right|_{{z = - \frac{{L_{1} }}{2}}} = 0} & {\left. w \right|_{{z = \frac{{L_{1} }}{2}}} = 0} \\ {\left. {\psi_{\varphi } } \right|_{{z = - \frac{{L_{1} }}{2}}} = 0} & {\left. {\psi_{\varphi } } \right|_{{z = \frac{{L_{1} }}{2}}} = 0} \\ \end{array}$$

**Figure 5:**
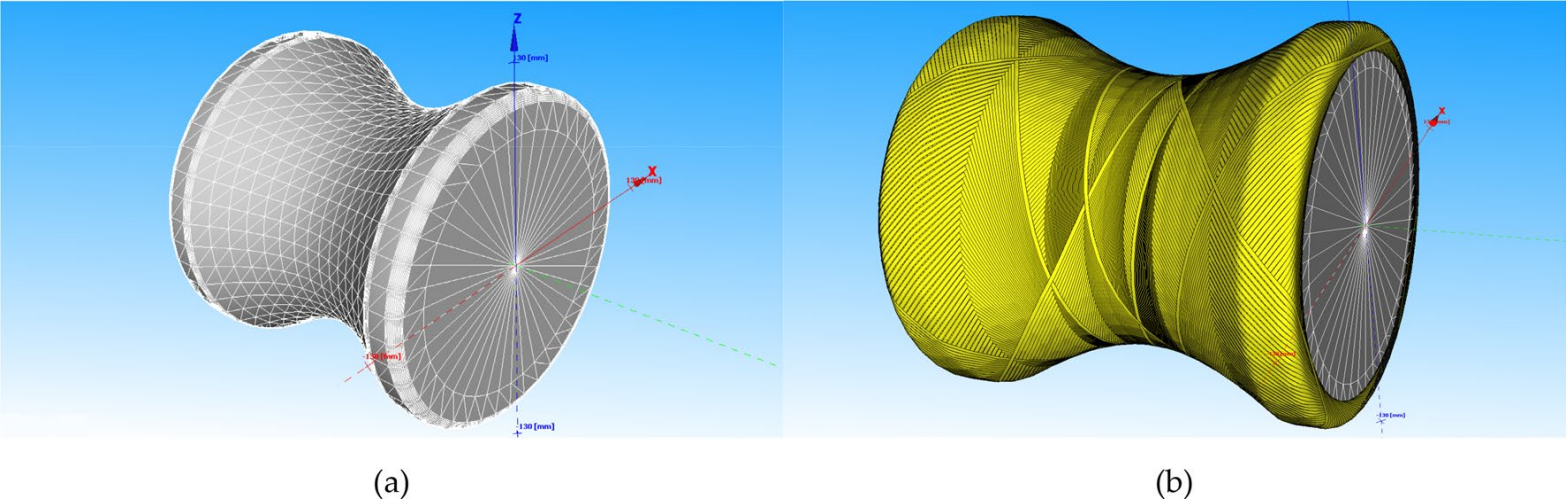
(**a**) The finite element model of reinforced layer. (**b**) The non-geodesic winding pattern with the initial winding angle of 52.5 degrees. Both figures were created with CADWIND v10. URL: https://www.material.be/cadwind/intro/.

The deformation of flexible pipes is mainly determined by the anisotropy of the reinforced layer. Therefore, it is important to establish the non-geodesic winding pattern accurately to simulate the anisotropy of the reinforced layer. The winding pattern is obtained with a given initial winding angle in software CADWIND, as shown in Fig. 5.

**Figure 6:**
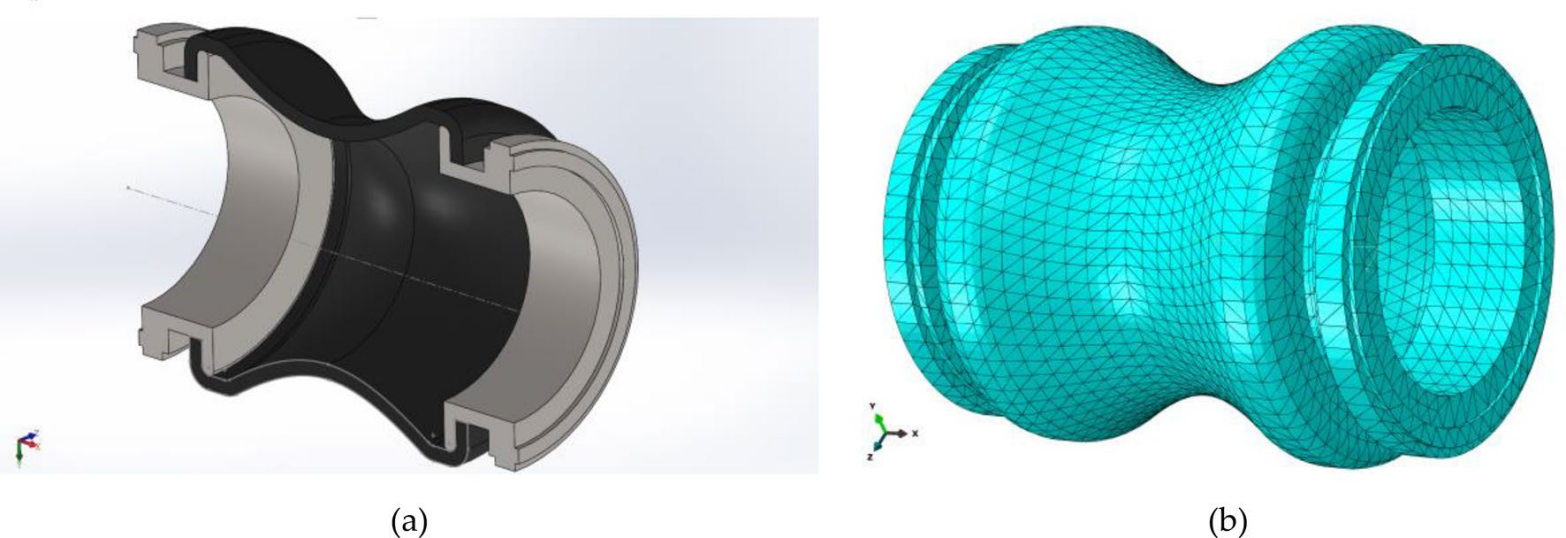
(**a**) The 3D model of the hyperbolic flexible pipe. The figure was created with SolidWorks 2016. URL: https://www.solidworks.com/ (**b**) The finite element model of the hyperbolic flexible pipe. The figure was created with ABAQUS 6.14. URL: https://www.3ds.com/products-services/simulia/products/abaqus/.

The models of the metal flanges and the flexible section are constructed with software SolidWorks and imported to software ABAQUS to perform the finite element analysis, as shown in Fig. 6a. The reinforced layer is imported from CADWIND to ABAQUS with an “embedded” constraint, as shown in Fig. 6b. The anisotropy of the reinforced layer is obtained with winding angle distribution calculated accurately in CADWIND. In the model definition file, several material sections are defined perpendicular to the axis of rotation. The winding angle of each material section is defined according to the winding angle distribution calculated in CADWIND. The mesh information of the finite element model is shown in Table 2. To calculate the deformation of the pipe, the global size of the pipe is 10 mm. The thickness of the reinforced layer is 1.2 mm, while the minimum circumferential diameter of the pipe is 150 mm. It is suitable to modeling the reinforced layer with the S4R element.

**Table 2:** Mesh information of the finite element model.

Part	Element type	Number of elements	Number of nodes	Global size (mm)
Flange	C3D10	10,966	19,956	12
Rubber layer	C3D10	11,666	22,489	10
Reinforced layer	S4R	1932	1974	10

## Results of the theoretical model and the finite element model

**Figure 7:**
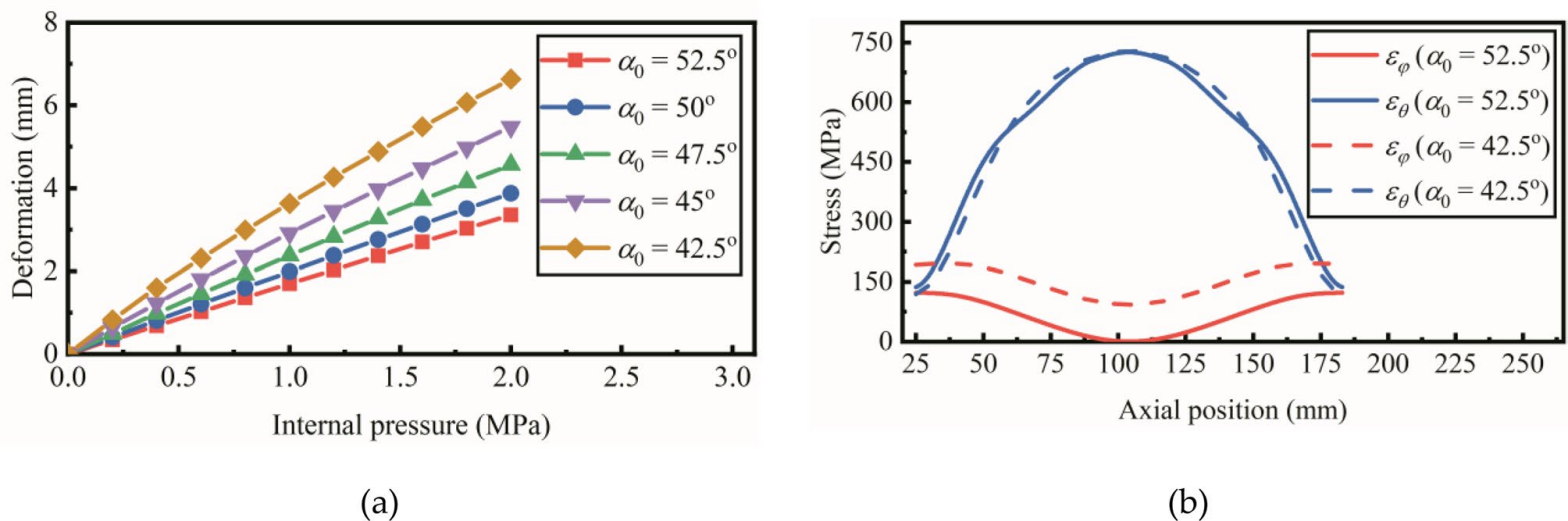
(**a**) Maximum deformation vs. the internal pressure with initial winding angle *α*0 increased from 42.5 degrees to 52.5 degrees. (**b**) The inplane stress distribution of the reinforced layer while the internal pressure is 1.0 MPa and the initial winding angle is 52.5 degrees and 42.5 degrees.

The relation between maximum deformation and internal pressure is shown in Fig. 7a. The initial winding angle *α*_0_ is increased from 42.5 degrees to 52.5 degrees. The maximum deformation is decreased significantly with a larger initial winding angle. The distribution of inplane stress *ε*_*φ*_ and *ε*_*θ*_ is calculated, as shown in Fig. 7b. The internal pressure is 1.0 MPa and the initial winding angle is 42.5 degrees and 52.5 degrees. At the middle of the pipe, the meridional stress (*ε*_*φ*_) is decreased to the minimum while the circumferential stress (*ε*_*θ*_) reaches the maximum. With a larger initial winding angle, the meridional stress is decreased significantly.

To improve the stability of the hyperbolic pipe, the winding trajectory is better to match the direction of the major principal stress. If the winding angle is equal to the direction of the major principal stress precisely, the following equation will be satisfied:

29 $${\text{cot}}^{2} \alpha - \frac{{\varepsilon_{\varphi } }}{{\varepsilon_{\theta } }} = 0$$

**Figure 8:**
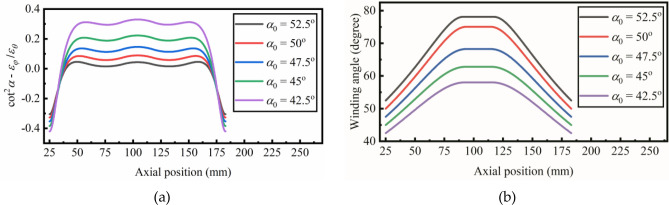
(**a**) The results of Eq. (29) with the initial winding angle increased from 42.5 degrees to 52.5 degrees. (**b**) The distribution of winding angle with the initial winding angle increased from 42.5 degrees to 52.5 degrees.

As shown in Fig. 8a, Eq. (29) is calculated with different initial winding angle. When the initial winding angle is increased from 42.5 degrees to 52.5 degrees, the variance between ${\mathrm{cot}}^{2}\alpha$ and ${\varepsilon }_{\varphi }/{\varepsilon }_{\theta }$ is closer to zero. The distribution of winding angle with different initial winding angle is illustrated in Fig. 8b. It could be concluded that large winding angle distribution reduces the meridional stress and improves the stability of the hyperbolic pipe.

**Figure 9:**
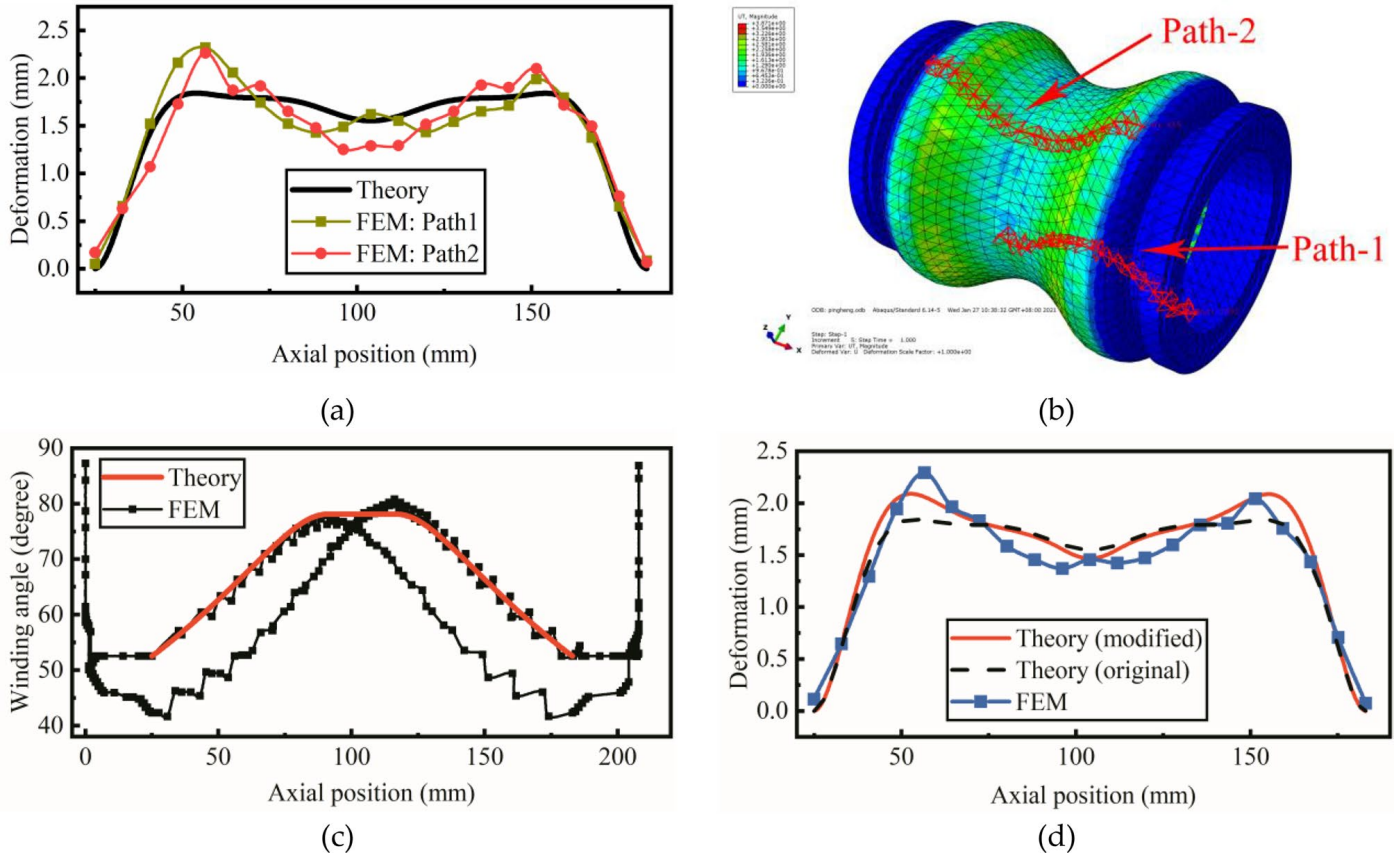
(**a**) The comparison of the deformation between the theoretical model and the finite element model. (**b**) The position of Path-1 and Path-2 on the finite element model. The figure was created with ABAQUS 6.14. URL: https://www.3ds.com/products-services/simulia/products/abaqus/ (**c**) The comparison of optimal winding angle between the theoretical model and the finite element model. (**d**) The comparison of the deformation among the original theoretical model, modified theoretical model and the finite element model. The FEM results is an anverage of the results of Path-1 and Path-2.

The comparison of the deformation between the theoretical model and the finite element model is shown in Fig. 9a. The initial winding angle is 52.5 degrees and the internal pressure is 1.0 MPa. The results of the FEM are calculated on two paths. Figure 9b shows the position of the path-1 and path-2 on the pipe. The maximum deformation of the theoretical model is 1.85 mm, while the maximum deformation of the finite element model is 2.30 mm. The relative error is 19.6%. Both models show that the maximum deformation occurs at the quarter of the hyperbolic pipe.

The relative error mainly comes from the variance of winding angle calculated in the two models, as shown in Fig. 9c. The black line is the wing angle of the complete winding trajectory simulated in CADWIND. The theoretical winding angle is in good agreement with the half trajectory of the simulated result. To form a complete winding trajectory, the winding angle of the other half trajectory has shifted a little from the theoretical result. In Fig. 9d, the red line shows the deformation calculated in the theoretical model with winding angle simulated in CADWIND. The maximum deformation is 2.1 mm and the relative error is decreased from 19.6% to 8.7%. The blue line is the average deformation of the results of Path-1 and Path-2.

## Discussion

**Figure 10:**
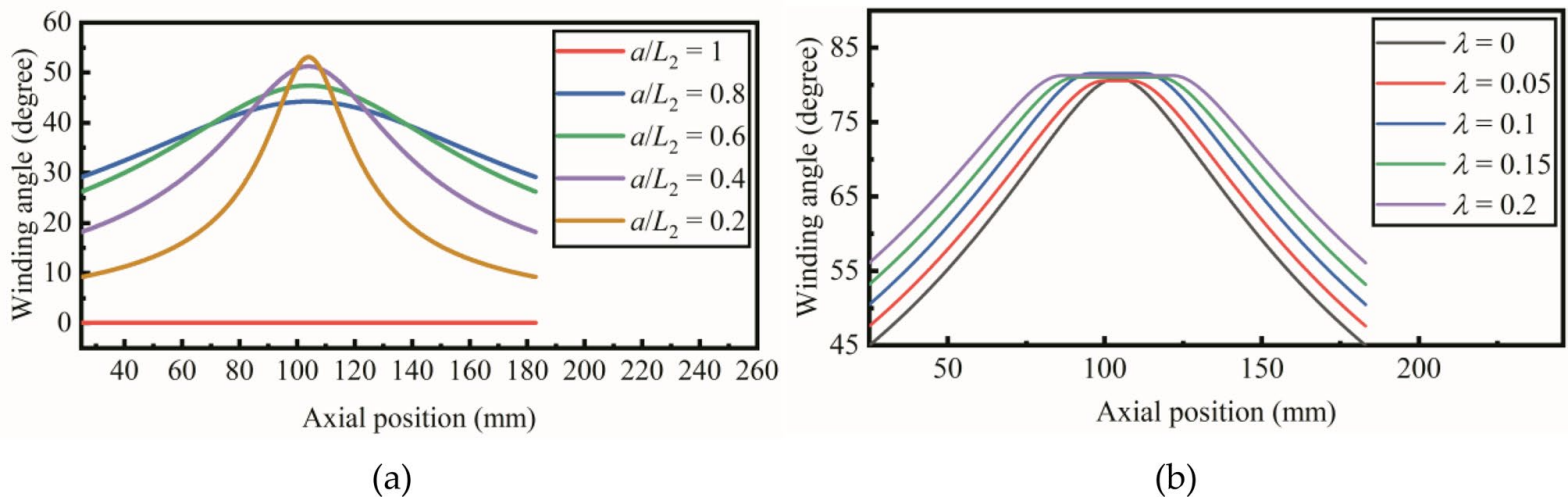
(**a**) The lower limit of available winding trajectoreis with different *a*/*L*2. (**b**) The upper limit of the initial winding angle with different slippage coefficient.

The available initial winding angle is limited by the geometry of the mandrel and the slippage coefficient. According to Eq. (10), the lower limit is determined by the ratio of the meridional radius *R*_*φ*_ and the circumferential radius *R*_*θ*_. To avoid the fiber bridge effect, the lower limit of available winding trajectories is calculated with different *a*/*L*_2_, as shown in Fig. 10a. The *L*_2_ represents the circumferential radius at both ends, while the *a* represents the circumferential radius at the middle of the pipe. When the *a*/*L*_2_ equals one, the hyperbolic pipe is transformed into a cylindrical pipe. The fiber bridge effect does not exist on cylindrical pipe, so there is no lower limit. The upper limit of the initial winding angle is determined by the slippage coefficient, as shown in Fig. 10b. The winding trajectory becomes geodesic when the slippage coefficient equals zero. The upper limit is increased from 45 to 56 degrees when the slippage coefficient is increased from 0 to 0.2.

**Figure 11:**
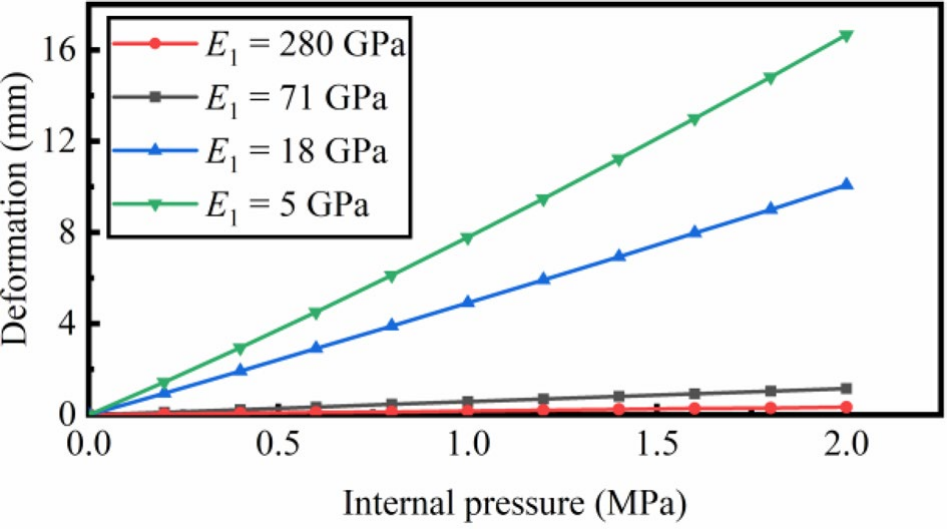
The maximum deformation vs. the internal pressure with variant reinforced fibers (Polyamide 66 – 5 GPa; Polyester– 18 GPa; Kevlar 29 – 71 GPa; Zylon – 280 GPa).

The reinforced fiber of high tensile modulus will reduce the deformation of the pipe. The relation between the maximum deformation and internal pressure is calculated with four different reinforced fiber, as shown in Fig. 11 and Table 3. The deformation is negligible when the PBO is used as reinforced fibers. The flexible pipe with the fiber of low tensile modulus suffers more severe deformation under internal pressure.

**Table 3:** The tensile modulus of the reinforced fibers^24^.

Fiber	Composition	Tensile modulus *E*_1_ (GPa)
Polyamide 66	PA66	5
Polyester	PET	18
Kevlar 29	PPTA	71
Zylon	PBO	280

**Figure 12:**
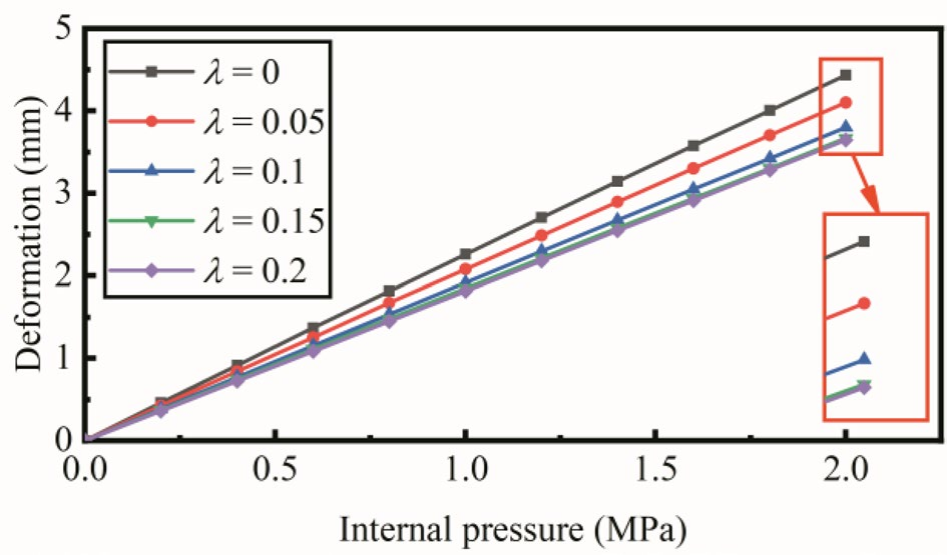
Maximum deformation vs. the internal pressure with the slippage coefficient increased form 0 to 0.2.

Compared with the geodesic winding trajectories (*λ* = 0), it indicates that the non-geodesic winding trajectories improve the stability of the hyperbolic pipe. Figure 12 shows the relation between maximum deformation and internal pressure with an increased slippage coefficient. The deformation reduces when the slippage coefficient *λ* increases from 0 to 0.15. However, when the slippage coefficient is larger than 0.15, the maximum deformation remains the same.

## Conclusion

The stability of filament-wound hyperbolic flexible pipe under internal pressure is achieved with optimal winding trajectory. Large winding angle distribution reduces the meridional stress and the deformation of the hyperbolic pipe. The lower limit of the initial winding angle is determined by the geometry of flexible pipes, and the upper limit is determined by the slippage coefficient. The reinforced filament of high tensile modulus will reduce the deformation of the pipe. Compared with the geodesic winding trajectory, non-geodesic winding trajectories will improve the stability of the pipe.
